# Focal adhesion kinase-mediated signaling controls the onset of pancreatic cell differentiation

**DOI:** 10.1242/dev.200761

**Published:** 2022-09-09

**Authors:** Uylissa A. Rodriguez, Shakti Dahiya, Michelle L. Raymond, Chenxi Gao, Christina P. Martins-Cargill, Jon D. Piganelli, George K. Gittes, Jing Hu, Farzad Esni

**Affiliations:** 1 Department of Surgery, Division of Pediatric General and Thoracic Surgery, Children's Hospital of Pittsburgh, University of Pittsburgh Medical Center, Pittsburgh, PA 15244, USA; 2 Department of Medicine, Division of Gastroenterology, Hepatology and Nutrition, University of Pittsburgh, Pittsburgh, PA 15244, USA; 3 Department of Developmental Biology, University of Pittsburgh, Pittsburgh, PA 15244, USA; 4 UPMC Hillman Cancer Center, Pittsburgh, PA 15123, USA

**Keywords:** Pancreas, Embryonic, FAK, Differentiation, Cell polarity

## Abstract

Signals from the endothelium play a pivotal role in pancreatic lineage commitment. As such, the fate of the epithelial cells relies heavily on the spatiotemporal recruitment of the endothelial cells to the embryonic pancreas. Although it is known that VEGFA secreted by the epithelium recruits the endothelial cells to the specific domains within the developing pancreas, the mechanism that controls the timing of such recruitment is poorly understood. Here, we have assessed the role of focal adhesion kinase (FAK) in mouse pancreatic development based on our observation that the presence of the enzymatically active form of FAK (pFAK) in the epithelial cells is inversely correlated with vessel recruitment. To study the role of FAK in the pancreas, we conditionally deleted the gene encoding focal adhesion kinase in the developing mouse pancreas. We found that homozygous deletion of *Fak* (*Ptk2*) during embryogenesis resulted in ectopic epithelial expression of VEGFA, abnormal endothelial recruitment and a delay in endocrine and acinar cell differentiation. The heterozygous mutants were born with no pancreatic phenotype but displayed gradual acinar atrophy due to cell polarity defects in exocrine cells. Together, our findings imply a role for FAK in controlling the timing of pancreatic lineage commitment and/or differentiation in the embryonic pancreas by preventing endothelial recruitment to the embryonic pancreatic epithelium.

## Abstract (precis)

**Summary:** Conditional inactivation of focal adhesion kinase in the developing pancreas reveals its role in controlling the timing of pancreatic lineage commitment and/or differentiation by preventing endothelial recruitment to the embryonic epithelium.

## INTRODUCTION

Focal adhesion kinase (FAK) is a cytoplasmic non-receptor tyrosine kinase that consists of three domains, each with independent functions (Lim et al., 2008). The N-terminal domain is responsible for binding FAK to growth factor receptors, cytokine receptors and G-protein-linked receptors. FAK is also involved in mediating integrin signaling (Schaller et al., 1992). Integrins engage with the extracellular matrix (ECM) and recruit FAK to form dynamic structures known as focal adhesions (Cai et al., 2012). The interaction between FAK and integrins is through its C-terminal domain (Yoon et al., 2015). Both the recruitment of FAK by integrins and FAK binding to the cytokine or to growth factor receptors promote autophosphorylation of FAK, which in turn activates the third domain, the FAK kinase domain, and triggers downstream signaling cascades that influence growth and differentiation (Cai et al., 2012; Lim et al., 2008; Mitra and Schlaepfer, 2006; Schlaepfer and Mitra, 2004; Tammela et al., 2017; Vadali et al., 2007; Yoon et al., 2015). In addition to its kinase-dependent activity, FAK can also complex with a number of proteins as a scaffold to enhance cell survival in a kinase-independent manner (Kleinschmidt and Schlaepfer, 2017; Yoon et al., 2015). FAK is expressed as early as gastrulation, and genetic deletions of FAK in mice result in embryonic lethality with defects in mesodermal development, specifically cardiovascular development (Corsi et al., 2006; Furuta et al., 1995; Ilić et al., 1995; Lim et al., 2010).

The pancreas is a highly branched organ. Noteworthy, tubulogenesis and branching of the epithelial cells in the embryonic pancreas are morphological events that not only help to shape the gland, but also control pancreatic cell specification (Azizoglu et al., 2017; Hick et al., 2009; Kesavan et al., 2014, 2009; Löf-Öhlin et al., 2017; Mamidi et al., 2018; Villasenor et al., 2010). The fate of embryonic pancreatic progenitor cells is influenced by their location within the embryonic anlagen long before any apparent lineage commitment. Cells residing in the core of the early pancreatic bud are called ‘body cells’, which later form the trunk of the pancreas (‘trunk’ cells) and are destined to become endocrine or ductal. On the other hand, cells in the periphery, the so-called ‘cap’ cells in the early pancreatic bud, and later the ‘tip’ cells, are initially multipotent cells, but later tend to differentiate into acinar cells (Shih et al., 2016; Zhou et al., 2007). As the body and cap cells are highly mobile, the lineage specification in the embryonic bud will be dictated by the extent to which a particular cell is exposed to the signals from the surrounding mesenchyme and ECM (Gittes, 2009; Kesavan et al., 2009; Shih et al., 2016). Proper pancreas development depends on a delicate balance between self-renewal and branching/differentiation of progenitor cells, as premature differentiation would deplete the progenitor pool and cause abnormal development (Magenheim et al., 2011). Several studies have highlighted the key role that endothelial cells or vessels play in maintaining this balance (Heymans et al., 2019; Magenheim et al., 2011; Pierreux et al., 2010). It is known that VEGFA, which is secreted specifically by trunk cells, recruits endothelial cells to the trunk area (Pierreux et al., 2010). Furthermore, endothelial-derived factors such as laminin promote endocrine differentiation among the bipotent trunk cells and promote commitment to the acinar lineage by the multipotent tip cells (Heymans et al., 2019; Kesavan et al., 2009; Pierreux et al., 2010).

Epithelial branching in the developing pancreas relies on ECM-integrin signaling, a process that is partially mediated by FAK (Mamidi et al., 2018; Shih et al., 2016). Accordingly, pharmaceutical inhibition of the kinase activity of FAK results in impaired branching of the cultured embryonic explants (Shih et al., 2016). Interestingly, this inhibition of FAK activity also promotes endocrine differentiation in cultured mouse pancreatic explants through expansion of Ngn3-expressing endocrine precursor cells (Mamidi et al., 2018). Similarly, treatment of human pluripotent stem cells with an FAK inhibitor seems to favor endocrine specification (Afrikanova et al., 2011). Together, these data suggest that FAK may be involved in pancreatic cell fate specification during development. In our study, conditional inactivation of FAK in the developing pancreas allowed us to study the function of FAK in pancreatic organogenesis.

## RESULTS

### pFAK expression in the pancreas

**Fig. 1.**
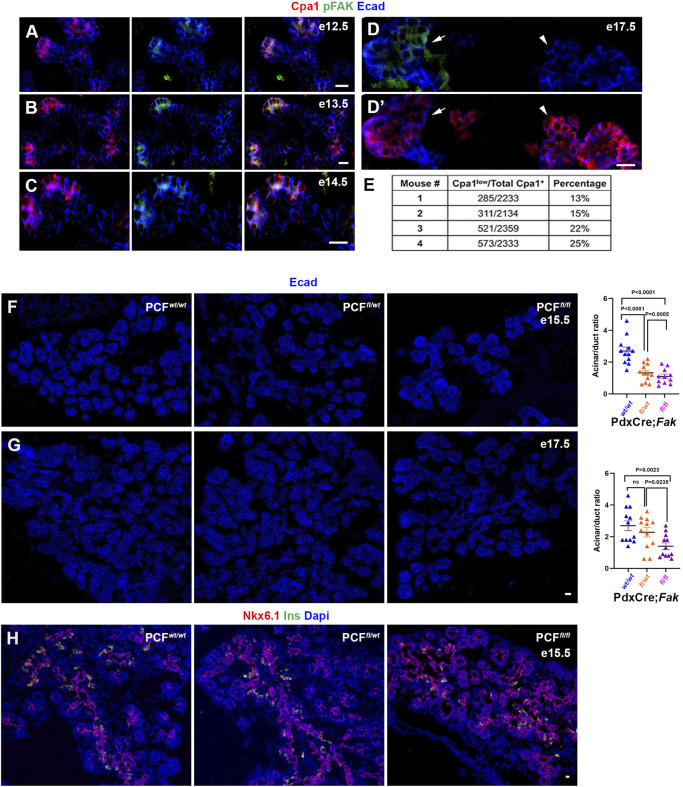
**pFAK is detected in all acinar precursors and is required for proper branching of pancreatic epithelium.** (A-D) Immunostaining for detection of Cpa1, pFAK and E-cadherin in embryonic wild-type pancreas at days 12.5 (*n*=5) (A), 13.5 (*n*=5) (B), E14.5 (*n*=5) (C) or E17.5 (*n*=5) (D) showing specific presence of pFAK in tip cells (A-C) or in a subset of acinar cells (D,D′). Arrows and arrowheads in D and D′ highlight pFAK^+^/CPA1^low^ (arrows) and pFAK^−^/Cpa1^hi^ (arrowheads) cells in the E17.5 pancreas. (E) Quantification of the percentage of Cpa1^low^ cells in the E17.5 pancreas. (F,G) Immunostaining and quantification of sections obtained from E15.5 (F) PCF*^wt/wt^* (*n*=4), PCF*^fl/wt^* (*n*=3) or PCF*^fl/fl^* (*n*=3), or E17.5 (G) PCF*^wt/wt^* (*n*=4), PCF*^fl/wt^* (*n*=4) or PCF*^fl/fl^* (*n*=4) pancreas for detection of E-cadherin, showing impaired branching and decreased acinar-to-ductal ratio in the mutant pancreas. (H) Immunostaining of sections obtained from E15.5 PCF*^wt/wt^* (*n*=4), PCF*^fl/wt^* (*n*=3) or PCF*^fl/fl^* (*n*=3) pancreas for detection of Nkx6.1. The persistent expression of Nkx6.1 in the mutant tip cells. Mann–Whitney *U*-test (Wilcoxon Rank Sum test). Data are mean±s.e.m. Scale bars: 20 μm.

The phosphorylated form of FAK (pFAK) is the enzymatically active molecule. In the developing pancreas, pFAK is first detected in the cap cells, which are in direct contact with the embryonic mesenchyme and ECM surrounding the pancreatic bud (Shih et al., 2016). Our immunostaining analyses revealed that at the time of acinar cell specification between embryonic day (E) 12 and E14, pFAK could be found exclusively in the carboxypeptidase A1^+^ (Cpa1^+^) cells, with Cpa1 being a marker of the tip cells (Fig. 1A-C). By E17.5, Cpa1 becomes a marker of maturing acinar cells rather than tip cells. Although most of the acinar cells in the E17.5 pancreas expressed high levels of Cpa1, a subpopulation (∼18%) displayed lower Cpa1 expression (Fig. 1D′,E). Interestingly, these Cpa1^low^ cells appeared to also be pFAK^+^ (Fig. 1D,D′). By P1 these Cpa1^low^/pFAK^+^ cells were not found (data not shown).

### FAK is required for proper branching during pancreatic development

In order to study the potential role of FAK in the developing pancreas, we generated the PdxCre;*Fak^fl/fl^* mice (PCF*^fl/fl^*) to specifically delete the gene encoding FAK in all pancreatic cells. At E15.5, the pancreas of PdxCre;*Fak^fl/wt^* heterozygous (PCF*^fl/wt^*) and PCF*^fl/fl^* mutant embryos displayed impaired branching and ∼50% lower acinar-to-ductal cell ratio than the littermate controls PdxCre;*Fak^wt/wt^* (PCF*^wt/wt^*) (Fig. 1F). Although this phenotype persisted in the E17.5 PCF*^fl/fl^* embryos, the pancreas of heterozygous mutant embryos was morphologically similar to the littermate controls with normal branching (Fig. 1G). The transcription factor Nkx6.1 is initially present in all pancreatic progenitor cells, including both trunk and tip cells, but later becomes excluded from Ptf1a-expressing cells upon acinar differentiation (Schaffer et al., 2010). The observed decreased acinar-to-ductal cell ratio in the mutant pancreas was associated with sustained expression of Nkx6.1 in the tip domain (Fig. 1H).

**Fig. 2.**
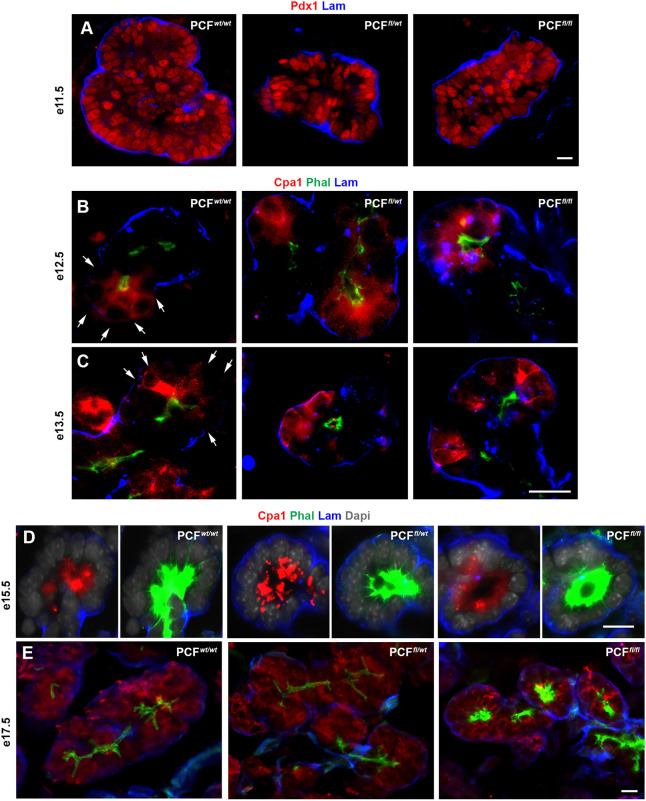
**Abnormal laminin deposition in the PdxCre;*Fak^fl/fl^* embryonic pancreas.** (A) Immunostaining for detection of Pdx1 and laminin in the E11.5 PCF*^wt/wt^* (*n*=5), PCF*^fl/wt^* (*n*=3) or PCF*^fl/fl^* (*n*=3) pancreas. (B-E) Immunostaining for detection of Cpa1, phalloidin and laminin on sections obtained from E12.5 (B) PCF*^wt/wt^* (*n*=4), PCF*^fl/wt^* (*n*=4) or PCF*^fl/fl^* (*n*=3); E13.5 (C) PCF*^wt/wt^* (*n*=4), PCF*^fl/wt^* (*n*=4) or PCF*^fl/fl^* (*n*=3); E15.5 (D) PCF*^wt/wt^* (*n*=4), PCF*^fl/wt^* (*n*=3) or PCF*^fl/fl^* (*n*=3); or E17.5 (E) PCF*^wt/wt^* (*n*=4), PCF*^fl/wt^* (*n*=4) or PCF*^fl/fl^* (*n*=4) pancreas showing abnormal laminin deposition in the PCF*^fl/fl^* pancreas. Arrows in B and C indicate the laminin-free areas surrounding a tip. Scale bars: 20 μm.

Given the role of FAK-mediated ECM-integrin signaling in branching of the developing pancreas, we studied the deposition of laminin (an integrin ligand) in the mutant pancreas. In accordance with previous studies (Heymans et al., 2019; Shih et al., 2016), we could detect a layer of laminin covering the E11.5 dorsal bud in all three genotypes (Fig. 2A). In E12.5-E15.5 PCF*^wt/wt^* embryos, the trunk cells but not the Cpa1^+^ tip cells were covered by laminin (Fig. 2B-D). Similar to the control embryos, laminin was initially lost around the Cpa1^+^ tip cells in E12.5 PCF*^fl/wt^* and PCF*^fl/fl^* pancreas (Fig. 2B). However, in contrast to the PCF*^wt/wt^* embryos, the entire Cpa1^+^ area in E13.5 and E15.5 PCF*^fl/wt^* and PCF*^fl/fl^* mutant pancreas was covered by laminin (Fig. 2C,D). By E17.5, however, along with the expected loss of pFAK in terminally differentiated acinar cells, laminin could be detected surrounding the acini, suggesting that the acini had matured (Fig. 2E). To find out whether the premature coverage by laminin in the mutant pancreases was indicative of defects in cell polarity, we next looked at the distribution of other cell polarity markers.

**Fig. 3.**
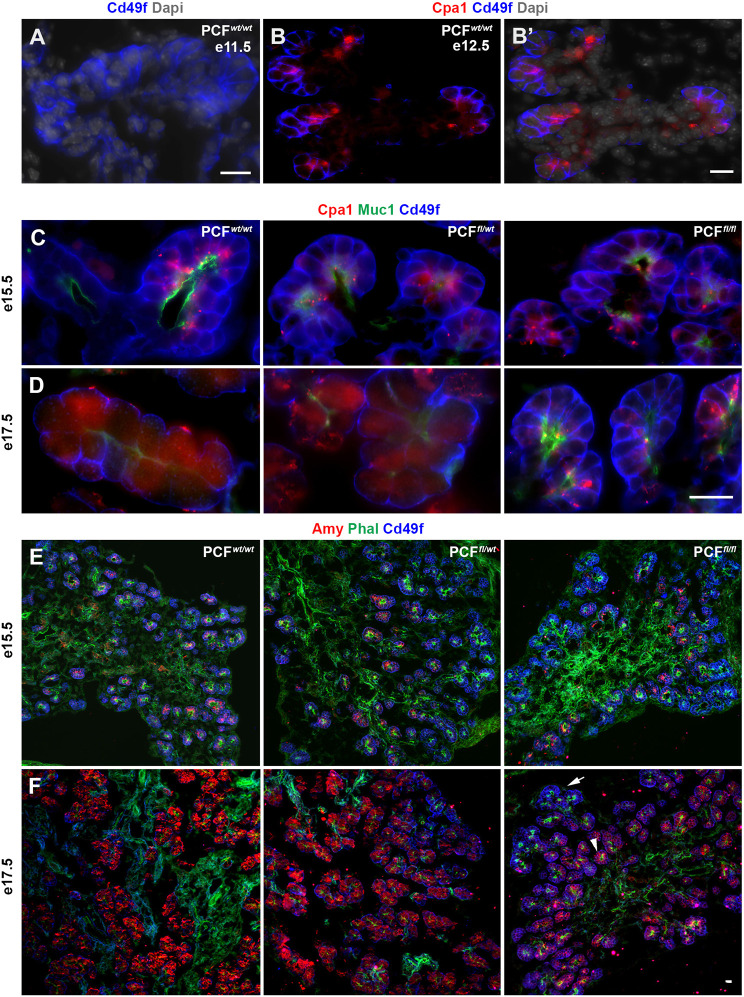
**Abnormal polarization and delayed acinar differentiation is observed in the PdxCre;*Fak^fl/fl^* pancreas.** (A,B) Immunostaining for detection of Cd49f (A), or Cpa1 and Cd49f (B) in the E11.5 (*n*=5) or E12.5 (*n*=5) pancreas showing Cd49f expression exclusively in the Cpa1^+^ tip cells. (C-F) Immunostaining for detection of Cpa1, Muc1 and Cd49f (C,D) or amylase, phalloidin and Cd49f (E,F) on sections obtained from E15.5 (C,E) PCF*^wt/wt^* (*n*=4), PCF*^fl/wt^* (*n*=3) or PCF*^fl/fl^* (*n*=3), or E17.5 (D,F) PCF*^wt/wt^* (*n*=4), PCF*^fl/wt^* (*n*=4) or PCF*^fl/fl^* (*n*=4) pancreas showing delayed acinar differentiation in the PCF*^fl/fl^* pancreas. Arrow in F indicates an amylase^−^ cluster with basolateral distribution of Cd49f. Arrowhead in F indicates an acinar cluster expressing amylase with Cd49f basally localized. Scale bars: 20 μm.

In PCF*^wt/wt^* embryos, as expected, Cd49f (integrin subunit α6) was found basolaterally in all pancreatic cells at E11.5 (Fig. 3A). However, by E12.5, Cd49f expression became restricted to the Cpa1^+^ acinar lineage (Fig. 3B,B′). Regardless of the genotype, Cd49f still showed a basolateral distribution at E15.5 (Fig. 3C and Fig. S1A). In PCF*^wt/wt^* and PCF*^fl/wt^* embryos, by E17.5, Cd49f could be found predominantly only on the basal side of the mature acinar cells (in proximity to the laminin), whereas its distribution persisted basolaterally in the PCF*^fl/fl^* acinar cells (Fig. 3D and Fig. S1B), suggesting a defect in acinar cell polarization. We next studied phalloidin, Muc1 and ZO1 as markers of acinar cell polarization. Between E12.5 and E15.5, all three genotypes showed the expected apical localization and high intensity staining for phalloidin (Figs 2B-D and 3E). By E17.5, however, the PCF*^wt/wt^* and PCF*^fl/wt^* embryos showed a dramatic decrease in intensity of staining, whereas the PCF*^fl/fl^* acinar cells at E17.5 still displayed the more immature high intensity staining seen at E15.5 (Figs 2E and 3F). For Muc1 (another marker of acinar cell polarization), the intensity pattern was similar to ZO1 for PCF*^wt/wt^* and PCF*^fl/wt^* embryos, but the PCF*^fl/fl^* acinar cells showed a lag in intensity such that, again, the E17.5 embryos appeared similar to E15.5 PCF*^wt/wt^* and PCF*^fl/wt^* embryos (Fig. 3C,D and Fig. S1C,D). Overall, the abnormal branching and gene expression pattern observed in the FAK-deficient pancreas indicates that FAK plays an important role in the developing pancreas, especially with regard to acinar maturation and polarization.

### FAK regulates the onset of acinar differentiation through endothelial recruitment to the tip cells

**Fig. 4.**
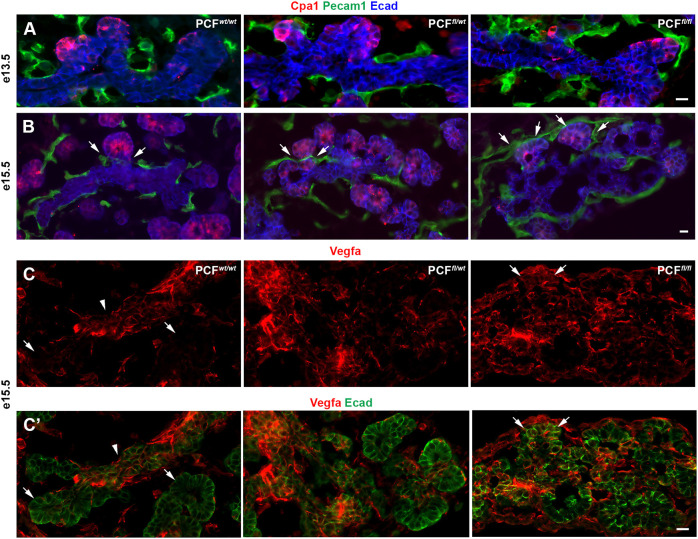
**Endothelial cells surrounding the tip region delay acinar differentiation in the PdxCre;*Fak^fl/fl^* pancreas.** (A-C′) Immunostaining for detection of Cpa1, Pecam and E-cadherin (A,B) or VEGFA and E-cadherin (C,C′) in E13.5 (A) PCF*^wt/wt^* (*n*=4), PCF*^fl/wt^* (*n*=4) or PCF*^fl/fl^* (*n*=3), and E15.5 (B-C′) PCF*^wt/wt^* (*n*=4), PCF*^fl/wt^* (*n*=3) or PCF*^fl/fl^* (*n*=3) pancreas showed premature endothelial cell recruitment to the Cpa1-expressing tip cells in the PCF*^fl/fl^* pancreas as a result of ectopic VEGFA expression in tip cells. Arrows in B mark endothelial cells appropriately surrounding trunk regions in PCF*^wt/wt^*, PCF*^fl/wt^* embryos, but inappropriately surrounding the tip domain in the PCF*^fl/fl^* pancreas. VEGFA is normally expressed in trunk cells. Arrows in C,C′ highlight the absence of VEGFA in tip cells in the control embryos and abnormal VEGFA expression in tip cells in the PCF*^fl/fl^* E15.5 pancreas. Arrowheads in C and C′ show VEGFA expression in trunk cells. Scale bars: 20 μm.

During early stages of pancreatic development, the trunk cells produce and secrete VEGFA, which results in specific recruitment of vessels to the trunk cells, but leaving the area surrounding the tip cells devoid of endothelial cells (Pierreux et al., 2010). The endothelial cells then deposit laminin around trunk cells (Heymans et al., 2019), which prevents epithelial branching and acinar differentiation (Magenheim et al., 2011; Pierreux et al., 2010; Sand et al., 2011). Given that tip cells in E13.5-E15.5 PCF*^fl/wt^* and PCF*^fl/fl^* pancreas were prematurely surrounded by laminin (Fig. 2C,D), we next stained for the endothelial marker Pecam1 (Cd31) to study the presence of endothelial cells within the pancreas in these embryos. As expected, only the trunk area in PCF*^wt/wt^* pancreas was surrounded by vessels, but not the tip cells (Fig. 4A,B). However, in the PCF*^fl/fl^* pancreas, the tip cells were completely surrounded by endothelial cells, whereas the PCF*^fl/wt^* embryos were intermediate between PCF*^wt/wt^* and PCF*^fl/fl^* embryos in that a fraction of Cpa1^+^ clusters were surrounded by vessels (Fig. 4A,B). The presence of endothelial cells around the tip cells in the PCF*^fl/fl^* pancreas was correlated with ectopic presence of VEGFA in these cells, (Fig. 4C,C′). Our finding indicates that VEGFA is downstream of FAK signaling in the tip cells. The premature presence of vessels correlates with the premature deposition of laminin that we observed (Fig. 2). Therefore, to study the effect of this premature laminin deposition on acinar differentiation, we looked for amylase expression at E15.5 and E17.5 (Fig. 3E,F and Fig. S1A,B). We found two distinct types of acinar clusters in the E17.5 PCF*^fl/fl^* pancreas. The first type of cluster was in the core of the embryonic pancreas, and, like their littermate controls, expressed amylase and displayed Cd49f basally. The second type of cluster, however, could be found mainly in the periphery of the pancreas, was amylase-negative and showed a basolateral distribution of Cd49f (Fig. 3E,F and Fig. S1A,B). These peripheral clusters were therefore less mature. Overall, these results indicate that FAK in the tip cells normally prevents premature recruitment of endothelial cells by suppressing VEGFA expression, and its absence is associated with apparently delayed acinar differentiation.

### Endocrine differentiation is affected in *Fak*-deficient embryos

**Fig. 5.**
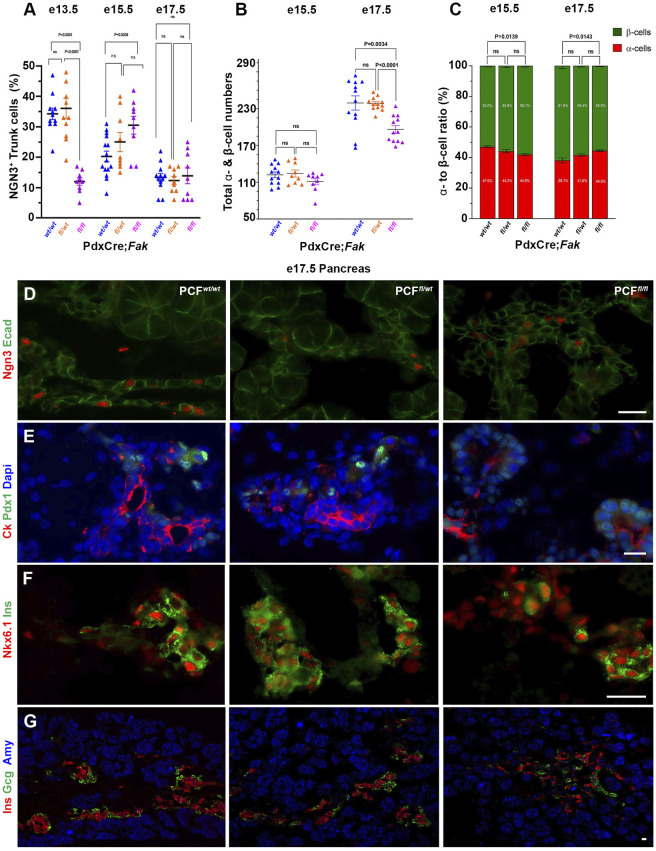
**Pancreatic endocrine differentiation is delayed in the PdxCre;*Fak^fl/fl^* embryos.** (A-C) Quantitative analysis comparing the percentage of Ngn3^+^ trunk cells (A), total number of α- and β-cells (B), and the ratio between α- and β-cells (C) in E13.5 (A) PCF*^wt/wt^* (*n*=4), PCF*^fl/wt^* (*n*=4) or PCF*^fl/fl^* (*n*=3); E15.5 (A-C) PCF*^wt/wt^* (*n*=5), PCF*^fl/wt^* (*n*=3) or PCF*^fl/fl^* (*n*=3); and E17.5 (A-C) PCF*^wt/wt^* (*n*=4), PCF*^fl/wt^* (*n*=3) or PCF*^fl/fl^* (*n*=3) pancreas. (D-G) Immunostaining of sections obtained from E17.5 PCF*^wt/wt^* (*n*=4), PCF*^fl/wt^* (*n*=4) or PCF*^fl/fl^* (*n*=4) pancreas for detection of E-cadherin and Ngn3 (D), Pdx1 and cytokeratin (E), insulin and Nkx6.1 (F), or glucagon, insulin and amylase (G). Endocrine specification is lagging behind in the PCF*^fl/fl^* embryos. Mann–Whitney *U*-test (Wilcoxon Rank Sum test). Data are mean±s.e.m. Scale bars: 20 μm.

We next decided to study the effect of *Fak* (*Ptk2*) deficiency on endocrine cells. To do so, we analyzed the expression of several transcription factors that are essential for proper pancreas development or endocrine lineage commitment and differentiation. Tip cells are characterized as Cpa1^+^, whereas the trunk domain is characterized by Ngn3^+^ cells (Gu et al., 2002; Zhou et al., 2007). Our analysis revealed a nearly threefold decrease and 1.5-fold increase in the number of Ngn3-expressing trunk cells in the E13.5 and E15.5 PCF*^fl/fl^* pancreas, respectively (Fig. 5A and Fig. S2A,B), reflecting a more immature status of the endocrine compartment as Ngn3^+^ cells peak at E13.5 and should be declining by E15.5. At this embryonic stage, expression or distribution of progenitor markers Pdx1 and Sox9 appeared normal in the mutant pancreas (Fig. S2C,D), whereas Nkx6.1 could be detected in both trunk and tip cells (Fig. 1H and Fig. S2E). Moreover, while there was no difference in the total number of hormone-positive endocrine cells among the three genotypes at E15.5, we found a higher α- to β-cell ratio in the PCF*^fl/fl^* embryos compared to the PCF*^wt/wt^* littermates, again suggesting a delay in endocrine differentiation (Fig. 5B,C and Fig. S2F,G).

By E17.5, Pdx1, Sox9 and Nkx6.1 expression in PCF*^wt/wt^* and PCF*^fl/wt^* embryos were excluded from the tip area, as expected (Fig. 5E,F and Fig. S2H). However, in E17.5 PCF*^fl/fl^* embryos, in accordance with the observed delayed acinar differentiation, we found inappropriate sustained expression of Pdx1 in tip cells (Fig. 5E). Furthermore, persistence of cells expressing Sox9 or Nkx6.1 indicated the delayed maturation of progenitor cells in PCF*^fl/fl^* embryos (Fig. 5F and Fig. S2H). At E17.5, the percentage of Ngn3-expressing trunk cells was similar in all three genotypes (Fig. 5A,B). However, the total number of α- and β-cells was 25% reduced in PCF*^fl/fl^* pancreas (Fig. 5B,G). In addition, the PCF*^fl/fl^* embryos persisted in having a higher α- to β-cell ratio than the other two genotypes (Fig. 5C,G). Together, these data suggest that both acinar and endocrine differentiation are delayed by FAK deficiency.

### FAK deficiency leads to a delay in pancreas development

**Fig. 6.**
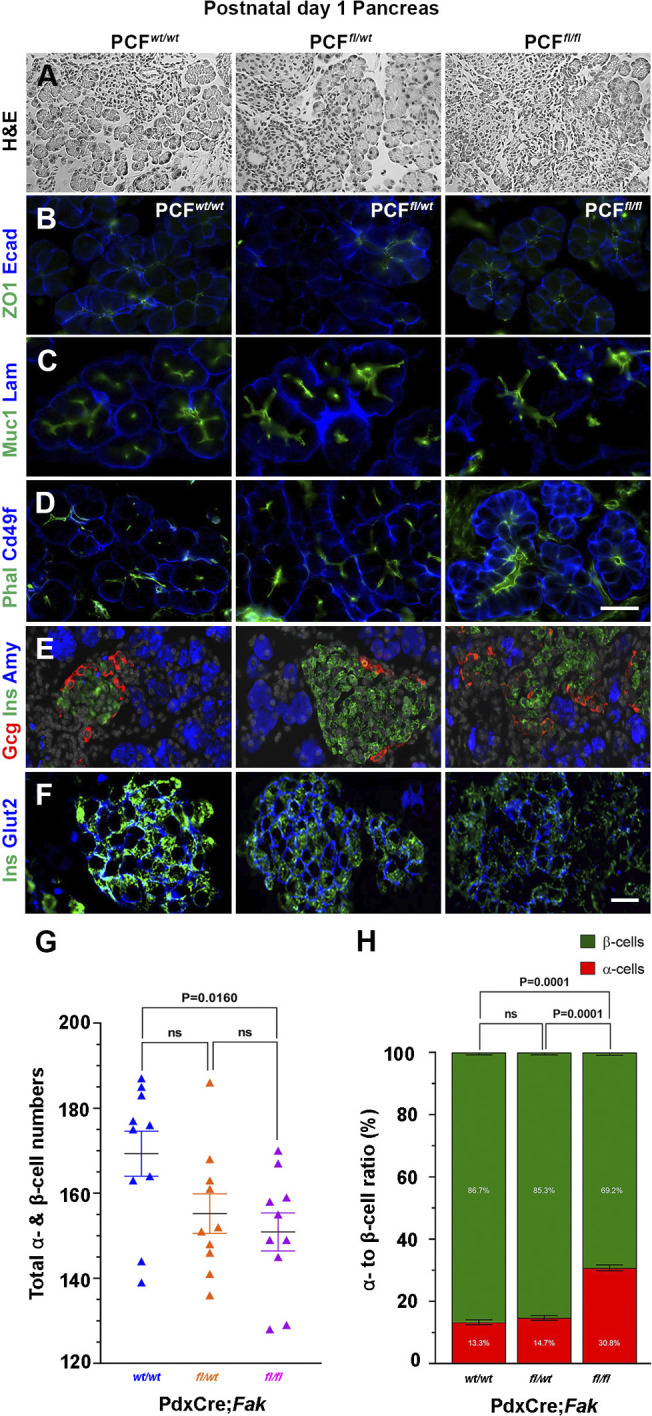
**Normal postnatal pancreatic tissue architecture and islet composition relies on FAK function.** (A-F) Hematoxylin and Eosin staining (A) or immunostaining of PCF*^wt/wt^* (*n*=5), PCF*^fl/wt^* (*n*=5) or PCF*^fl/fl^* (*n*=5) postnatal day 1 pancreas for detection of E-cadherin and ZO1 (B); Muc1 and laminin (C); phalloidin and Cd49f (D); amylase, insulin and glucagon (E); or Glut2 and insulin (F). There is abnormal intermixing of acinar and endocrine cells in A and E, and a subpopulation of acinar cells with basolateral distribution of Cd49f is present in the PCF*^fl/fl^* pancreas (D). (G,H) Quantitative analysis comparing total number of α- and β-cells (G), or the ratio between α- and β-cells (H) in PCF*^wt/wt^* (*n*=5), PCF*^fl/wt^* (*n*=5) or PCF*^fl/fl^* (*n*=5) postnatal day 1 pancreas. Kruskal–Wallis one-way analysis of variance (ANOVA) test. Data are mean±s.e.m. Scale bars: 20 μm.

Mice carrying pancreas-specific deletion of *Fak* were born at the expected Mendelian rate. Nevertheless, despite having similar body size to their control littermates, PCF*^fl/fl^* mice died within 24 h of birth. We next analyzed the mutant pancreas to determine whether the observed postnatal death was the result of a pancreatic phenotype. The postnatal day 1 (P1) *Fak*-deficient pancreas appeared grossly indistinguishable from the controls; however, histological analysis revealed an abnormal tissue architecture in the mutant pancreas. In PCF*^wt/wt^* and PCF*^fl/wt^* P1 pancreas, the endocrine islets were clearly separated from the adjacent acinar compartment, whereas in the PCF*^fl/fl^* pancreas acinar cells were intermixed with endocrine cells and showed an uneven distribution of amylase within the cytoplasm compared with the control acinar cells (Fig. 6A,E). At the cellular level, acinar cells from all three genotypes displayed normal apical ZO-1, Muc1 and phalloidin distribution, along with normal basal laminin localization (Fig. 6B-D). However, a subset of acinar cells in PCF*^fl/fl^* pancreas showed a basolateral distribution of CD49f (Fig. 6D), suggesting improper polarization.

Notably, although the PCF*^fl/fl^* islets to some extent maintained the typical peripheral α-cells and central β-cells, they formed more loosely aggregated clusters (Fig. 6E). This uncharacteristic islet structure was accompanied by the absence of glucose transporter 2 (GLUT2), which is normally expressed by mature β-cells, in a significant number of β-cells in the PCF*^fl/fl^* pancreas (Fig. 6F). Additionally, embryonic deletion of *Fak* reduced the total number of α- and β-cells in PCF*^fl/fl^* P1 pancreas (Fig. 6G). Furthermore, an approximate 5:1 ratio of β- to α-cells in the PCF*^wt/wt^* or PCF*^fl/wt^* P1 pancreas was reduced to 3:1 in PCF*^fl/fl^* mice, suggesting a more immature endocrine phenotype (Fig. 6H). Together, our data suggest that *Fak* deficiency results in delayed pancreatic differentiation and altered pancreatic architecture at P1.

### FAK is required for adult pancreatic acinar cell integrity and maintenance

The PCF*^fl/wt^* pups appeared healthy with no obvious pancreatic phenotype at P15. By P21, however, there were early signs of tissue damage, as reflected by periductal deposition of stroma and loss of the normal lobular acinar architecture (Fig. S3A,B). Moreover, we saw insulin-producing cells, either single or small clusters, with no detectable Glut2 expression (Fig. S3C,D). At P30, intact islets and small β-cell clusters were found primarily in close proximity to the larger ducts in the PCF*^fl/wt^* pancreas (Fig. S4A-C). Over the course of time, the PCF*^fl/wt^* acinar compartment became progressively atrophic, and by 1 year the pancreas consisted of areas with only a few acinar remnants, ducts and endocrine islets (Fig. S4D,E).

Given the excessive fibrotic tissue surrounding the ducts, we reasoned that the gradual loss of acinar tissue may be due to a ductal defect. We thus stained the tissues for cytokeratin and Muc1, which are known to mark exclusively the duct cells in the adult pancreas. In the PCF*^wt/wt^* pancreas, cytokeratin was uniformly detected in all duct cells, whereas the PCF*^fl/wt^* ducts displayed a patchy staining pattern (Fig. S4F,J). Moreover, while Muc1 was distributed on the apical side of all ducts in the control pancreas, it could be found only in the intercalated ducts in the PCF*^fl/wt^* pancreas (Fig. S4G,K). In addition to Muc1, the apical marker ZO1 was also absent in most duct cells in the mutant pancreas (Fig. S4H,L). In the PCF*^fl/wt^* acinar cells, ZO1 could be detected laterally rather than its normal apical localization (Fig. S4I,M). These results indicate that the observed acinar atrophy is likely to be the result of both a ductal defect and abnormal acinar polarity.

**Fig. 7.**
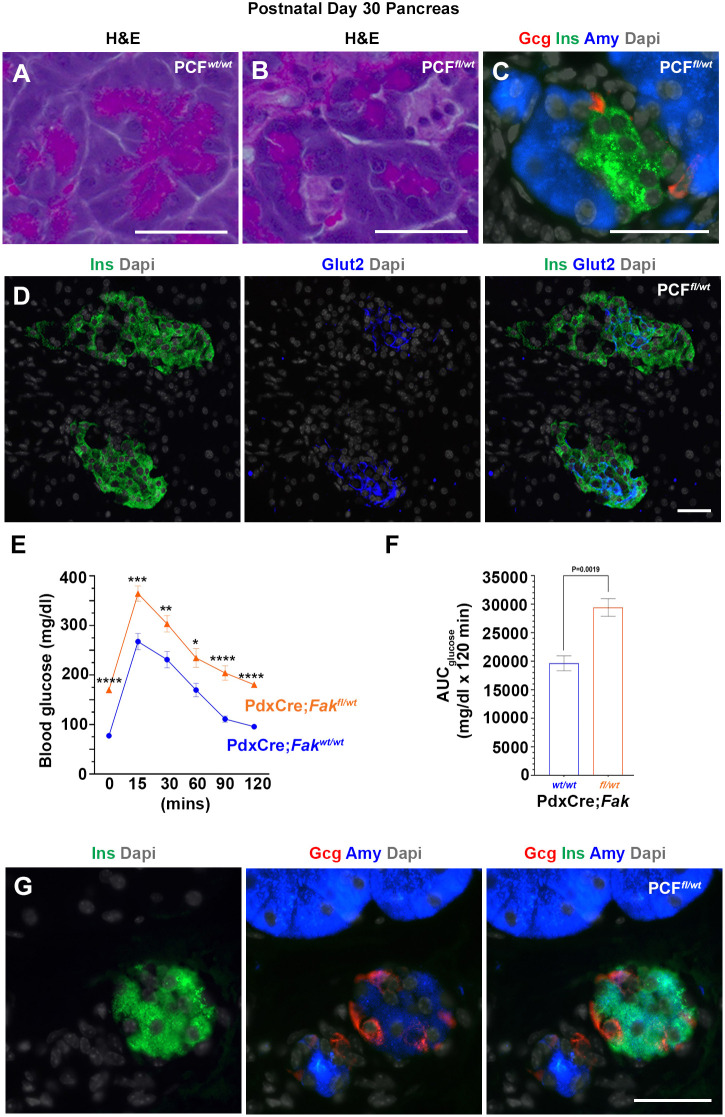
**Appearance of cells co-expressing amylase and insulin in PCF*^fl/wt^* mice.** (A-C) Hematoxylin and Eosin (A,B) or immunostaining for detection of insulin, glucagon and amylase (C) of P30 PCF*^wt/wt^* (*n*=3) (A) or PCF*^fl/wt^* (*n*=5) (B,C) pancreas showing acini composed of both amylase^+^ or insulin^+^ cells. (D) Immunostaining of P30 PCF*^fl/wt^* pancreas using antibodies against insulin and Glut2 showing insulin^+^/Glut2^−^ cells. (E,F) Glucose tolerance testing performed on 10-week-old PCF*^wt/wt^* (*n*=10), PCF*^fl/wt^* (*n*=10) mice (males and females) showing impaired glucose clearance in the PCF*^fl/wt^* mice. (G) Immunostaining of P30 PCF*^fl/wt^* (*n*=5) pancreas for detection of insulin, glucagon and amylase, showing a cluster of insulin^+^/amylase^+^ cells. Glucagon^+^/amylase^+^ cells are absent. *P=0.0124, **P=0.0062, ***P=0.0004, ****P<0.0001 in E. Unpaired Student's *t*-test in E; Dunnett's multiple comparisons test in F. Data are mean±s.e.m. Scale bars: 20 μm.

In the normal postnatal pancreas, each acinus consists of pyramidal-shaped acinar cells, which have a broad basal region and a narrow apical surface that surrounds a small central lumen (Fig. 7A). In the P30 PCF*^fl/wt^* pancreas, however, we found pancreatic acini that were composed of both amylase-positive cells and insulin-producing β-cells (Fig. 7B,C). In addition, we found that GLUT2 was detected in only a subpopulation of all β-cells in the PCF*^fl/wt^* mice (Fig. 7D). Accordingly, intraperitoneal glucose tolerance testing 10 weeks postnatally revealed both a higher fasting blood glucose and impaired glucose tolerance in PCF*^fl/wt^* mice when compared with the age- and sex-matched controls (Fig. 7E,F and Fig. S5A,B). We could not find any body weight differences among the cohorts (Fig. S5C). However, perhaps the most surprising finding in the PCF*^fl/wt^* pancreas was the presence of clusters of cells, although rarely found, that co-expressed amylase and insulin (Fig. 7G). Together, these data indicate that FAK is necessary for acinar maintenance, and that deletion of *Fak* leads to the appearance of cells co-expressing acinar and β-cell markers.

## DISCUSSION

Epithelial branching is a crucial process for the development of many tissues. In the embryonic pancreas, the epithelial cells are constantly and dynamically remodeled to build a functional organ. In fact, accumulating data show that cell specification in the developing pancreas relies on morphological changes in the epithelium (Azizoglu et al., 2017; Hick et al., 2009; Kesavan et al., 2009; Löf-Öhlin et al., 2017; Mamidi et al., 2018; Villasenor et al., 2010). Previous studies have shown that chemical inhibition of FAK kinase activity in embryonic pancreatic explants resulted in defects in epithelial branching (Mamidi et al., 2018; Shih et al., 2016), as well as enhanced endocrine differentiation in general and α-cell differentiation in particular (Mamidi et al., 2018). Given the involvement of FAK-mediated integrin signaling in both tubulogenesis and cell differentiation (Mamidi et al., 2018; Shih et al., 2016), we sought to study the role of FAK in pancreatic development *in vivo*.

Here, we have found that PCF*^fl/fl^* embryos display generally less well-developed branched pancreatic epithelium compared with the PCF*^wt/wt^* littermates. Interestingly closer examination of the localization of polarization markers laminin, Muc-1, Cd49f, phalloidin and ZO-1 revealed a similar distribution pattern for these markers when comparing the E17.5 mutant with the E15.5 wild-type pancreas. Given the normal distribution of the abovementioned cell polarity markers in the PCF*^fl/fl^* pancreas on postnatal day 1, it is likely that the observed embryonic phenotype in mutant pancreas is more the result of a delay in pancreas development rather than being due to impaired branching, as reported elsewhere (Mamidi et al., 2018; Shih et al., 2016). Pancreatic explant development *in vitro* tends to lag behind development of the embryonic pancreas *in utero* by 1-2 days, and generally reaches a maximum stage of development comparable with E15.5 pancreas *in utero* (Esni et al., 2005; Rhodes et al., 2012). Thus, the inherent limitation of the *in vitro* system, along with our observation that the branching phenotype in the mutants *in vivo* was a late event, and fairly subtle, may explain the discrepancies between the current and previous reports.

The E17.5 wild-type acinar compartment is composed of Cpa1^+^/amylase^+^ cells. Notably, in the E17.5 *Fak-*deficient pancreas, we could identify two distinct populations of Cpa1^+^ cells. The Cpa1^+^ cells in the periphery of the pancreas did not express amylase, whereas those located in the core of the pancreas were amylase^+^. Moreover, these amylase^−^ peripheral cells showed persistent expression of Pdx1, Sox9 and Nkx6.1, as well as localization of Cd49f to the basolateral region of the cells, resembling the Cpa1^+^ cells found at earlier developmental stages in the control pancreas. It has been demonstrated that between E10.5 and E13.5, Cpa1^+^ tip cells mark a multipotent cell population that can give rise to all pancreatic cell types, whereas by E13.5-15.5 they differentiate exclusively into acinar cells (Pan et al., 2013; Zhou et al., 2007). We show here that the transition of tip cells from a multipotent to a unipotent state coincides with a transient decrease in laminin deposition around the tip. This finding is consistent with the fact that laminin promotes commitment to the acinar lineage within multipotent cells (Kesavan et al., 2009), but at the same time can also suppress full acinar cell maturation (Heymans et al., 2019). Accordingly, the sustained presence of laminin around tip cells in the PCF*^fl/fl^* pancreas could prevent full acinar maturation by committed precursor cells. These data indicate that acinar lineage commitment, as evidenced by the presence of Cpa1^+^ cells, can proceed normally in the absence of functioning FAK; however, the subsequent terminal differentiation of these cells is halted in the PCF*^fl/fl^* embryos.

Cells within the trunk domain are bipotent progenitors that can contribute to the endocrine and ductal lineages (Zhou et al., 2007). Although exposure of these cells to laminin represses Ptf1a expression and conversely induces Ngn3 expression (Heymans et al., 2019; Mamidi et al., 2018), abnormal exposure of multipotent pancreatic cells to laminin has been reported to negatively impact endocrine differentiation (Kesavan et al., 2009). Given that in the *Fak*-mutant pancreas, abnormal laminin deposition was detected only around the tip area, it is unlikely that the higher ratio of Ngn3^+^ trunk cells would be due to the prolonged exposure to laminin. Furthermore, whereas the relative number of Ngn3^+^ cells peaks normally around E13-E14 (Villasenor et al., 2008), in the PCF*^fl/fl^* pancreas this number peaked at E15.5, which may reflect a delay in endocrine lineage selection.

Moreover, despite the increase in endocrine precursors at E15.5, we saw a decline in the total number of α- and β-cells, as well as a higher α- to β-cell ratio in the E17.5 and P1 PCF*^fl/fl^* pancreas. Interestingly, the α- to β-cell ratio in the mutant pancreas at any studied gestational time point resembled that of wild-type embryos at an earlier stage. Together, our findings suggest that both acinar and endocrine differentiation are delayed in the PCF*^fl/fl^* pancreas.

The PCF*^fl/fl^* mice died on postnatal day 1; however, the observed pancreatic phenotype is unlikely to be the reason for their death, as other mutants with no pancreas at birth can survive for several days (Jonsson et al., 1994; Offield et al., 1996). The exogenous *Pdx1* promoter used to express Cre-recombinase in this study has been shown to be active in different areas of the brain (Wicksteed et al., 2010). Thus, it is likely that *Fak* deletion in some part of the brain could cause the early death observed in PCF*^fl/fl^* mice.

Except for an abnormal laminin deposition, the PCF*^fl/wt^* pancreas looked generally indistinguishable from the control littermates. The PCF*^fl/wt^* pups appeared heathy and normal at birth, with no apparent pancreatic abnormalities. However, we observed a progressive loss of acinar tissue associated with defects in cell polarity in the exocrine cells. This finding is in line with other studies reporting that disrupted acinar cell polarity exposes the parenchyma to the digestive enzymes, which in turn causes acinar atrophy (Bombardelli et al., 2010; Hezel et al., 2008; Wang et al., 2014). Along with the exocrine phenotype, although nearly all β-cells in the P1 PCF*^fl/wt^* pancreas expressed Glut2, by P30 there were a large number of insulin^+^/Glut2^−^ cells. Accordingly, the PCF*^fl/wt^* mice had higher fasting blood glucose and displayed impaired glucose clearance. Given that mice with specific deletion of *Fak* in β-cells (insulin promoter driving Cre recombinase) have normal Glut2 levels (Cai et al., 2012), the absence of Glut2 in our study using a Pdx1 promoter suggests that FAK is specifically important for β-cell development.

Perhaps the most surprising finding in our study was the appearance of insulin^+^/amylase^+^ cells in P30 PCF*^fl/wt^* mice. Pdx1 is one of the earliest markers for pancreatic lineage; thus, the presence of cells co-expressing amylase and insulin in 1-month old PCF*^fl/wt^* mice could be due to abnormal pancreatic development in the mutant embryos. Alternatively, this phenomenon could be the result of transdifferentiation of acinar into β-cells, or vice versa. Future studies will reveal the exact underlying mechanism behind this apparent lineage conversion.

The role of endothelial-derived factors in promoting endocrine differentiation among the bipotent trunk cells and commitment to the acinar lineage by multipotent tip cells has been extensively studied (Heymans et al., 2019; Kesavan et al., 2009; Pierreux et al., 2010). However, what controls the timing of the endothelial recruitment is elusive. In this study, we show that the presence of pFAK in tip cells is most prominent between embryonic days 13 and 14, which coincides with the commitment of multipotent tip cells to the acinar lineage. Given that tip cells in the PCF*^fl/fl^* pancreas are covered by endothelial cells and laminin as early as at E13.5, we propose that pFAK prevents recruitment of endothelial cells to the tip cells by suppressing *Vegfa* expression. Our finding that VEGFA expression in tip cells is directly or indirectly regulated by FAK activity is different from other cell types, where phosphorylation of FAK appears to be downstream of VEGFA (Qi and Claesson-Welsh, 2001; Sun et al., 2018). Although the detailed mechanism for the causal relationship between pFAK and *Vegfa* expression in the developing pancreas remains unclear, our findings provide a model for the onset of acinar differentiation. Cells in the periphery of early pancreatic bud are exposed to ECM and laminin, which in turn generates a bias towards commitment to the acinar lineage. However, concomitant with the epithelial expansion, branching and the subsequent formation of the tip domains, laminin is gradually lost around the pFAK^+^/Cpa1^+^ tip cells. Once pFAK levels are decreased, endothelial cells responsible for laminin deposition are recruited to the tip cells, and terminal differentiation of acinar progenitors can be initiated.

## MATERIALS AND METHODS

### Mice

Mice used in these studies were maintained according to protocols approved by the University of Pittsburgh Institutional Animal Care and Use Committee. All breeding colonies were maintained under cycles of 12 h light and 12 h dark. Midday of the day the observed vaginal plug was considered as embryonic day (E) 0.5. The wild-type C57bl/6 mice were purchased from The Jackson Laboratory. The PdxCre (Criscimanna et al., 2011; Hingorani et al., 2003) and the *Fak*-floxed (Beggs et al., 2003) mice were obtained from the Mouse Models of Human Cancer Consortium (MMHCC) and the Mutant Mouse Resource and Research Centers (MMRRC), respectively.

### Immunohistology

Tissue processing and immunostaining were performed as previously described (Criscimanna et al., 2014, 2011; Socorro et al., 2017). Briefly, harvested pancreata were fixed overnight at 4°C in 4% paraformaldehyde, incubated in 30% sucrose solution overnight at 4°C and subsequently embedded with OCT compound. Sections were permeabilized with 0.1% PBS/Triton X-100, washed in PBS and blocked for 30 min in 10% normal donkey serum (NDS) in 0.1% PBS/Triton X-100. Primary antibodies were incubated overnight at 4°C, while secondary antibodies were incubated for 1 h at room temperature.

### Antibodies

The following antibodies were used: goat anti-amylase (1:250, Santa Cruz, sc-12821); rabbit anti-amylase (1:100, Sigma, A8273); rat anti-Cd31 (1:50, BD Bioscience, 550274); rat anti-Cd49f (1:100, BD Bioscience, 555734); rabbit anti-Cpa1 (1:100, Proteintech, 15836-1-AP); rabbit anti-cytokeratin (1:100, Dako, Z0622); goat anti-E-cadherin (1:200, R&D Systems, AF748); rabbit anti-glucagon (1:1000, Linco/Millipore, 4031-01F); goat anti-Glut2 (1:50, Santa Cruz, sc-31825); guinea pig anti-insulin (1:200, Abcam, ab195956); rabbit anti-insulin (1:500, Abcam, ab181547); rat anti-laminin (1:200, Origene, BM6046P); rabbit anti-MafA (1:200, Cell Signaling, 797373); Armenian hamster anti-Muc1 (1:100, Invitrogen, MAS-11202); rabbit anti-Ngn3 (1:100, Novusbio, NBP227115SS); rabbit anti-Nkx6.1 (1:300, Abcam, ab221549); goat anti-Pdx1 (1:7500, Abcam, ab47383); Alexa Fluor 488-conjugated phalloidin (1:500, Invitrogen, A12379); rabbit anti-pFAK (1:500, Invitrogen, 70025s); rabbit anti-Sox9 (1:10,000, Millipore, AB5535); rabbit anti-VEGFA (1:400, Abcam, ab52917); and rat anti-ZO1 (1:500, Millipore, MABT11).

All of the following secondary antibodies used for immunostaining were purchased from Jackson ImmunoResearch Laboratories: biotin-conjugated anti-rabbit (1:500, 711-066-152), biotin-conjugated anti-rat (1:500, 712-066-153), biotin conjugated anti-guinea pig (1:500, 706-065-148), biotin-conjugated anti-goat (1:250, 705-065-147); Cy2-conjugated streptavidin (1:500, 016-540-084); Cy3-conjugated streptavidin (1:500, 016-160-084); Cy5-conjugated streptavidin (1:100, 016-600-084); and Cy2- and Cy3- anti-guinea pig (706-545-148; 706-166-148), anti-rabbit (711-485-152; 711-165-152), anti-rat (712-545-153; 712-166-150) and anti-goat (705-545-147; 705-165-147) (all 1:300).

### Quantification analysis

To estimate the total number of α- and β-cells, or the percentage of Ngn3^+^ trunk cells, whole E13.5, E15.5, E17.5 or P1 pancreata were sectioned and immunostained. Sections (8-10 μm) were collected serially so that each slide would contain semi-adjacent sections across the entire tissue. Data were obtained by analyzing four to six sections per time point in four or five embryos or pups for each genotype. Quantification of cell number was performed using ImageJ software.

### Imaging

Imaging of pancreatic tissue sections was performed using a Leica Dmi8 fluorescent light microscope at 10×, 20× or 63× objectives using LASX software. The microscope is equipped with 405, 488, 568 and 647 nm filters. Final figures were composed using Adobe Photoshop.

#### Intraperitoneal glucose tolerance test (GTT)

Overnight 16-h-fasted mice were injected intraperitoneally with 2 g/kg glucose (Sigma-Aldrich). Blood glucose was measured from the tail vein at 0, 15, 30, 60, 90 and 120 min after injection using a glucometer (Contour NEXT EZ).

### Statistical analysis

Statistical analysis was performed using GraphPad Prism 9 software (GraphPad software version 9.2.0). Mann–Whitney *U* tests (Wilcoxon Rank Sum test) were used to evaluate differences between individual groups at their specific timepoints. Differences between genotypes of the same strain were assessed using a Kruskal–Wallis one-way analysis of variance (ANOVA), Dunnett's multiple comparisons test and a Bartlett's test for equal variance. Statistical significance between groups was accepted at the *P*<0.05 level. Unless specified, data in the text, table and figures are expressed as a mean±s.e.m.

## Supplementary Material

Supplementary information
