# Supplementary material for: Focal adhesion kinase-mediated signaling controls the onset of pancreatic cell differentiation
Source: Development. 2022 Sep 9;149(17):dev200761. doi: 10.1242/dev.200761 (PMC9482336; doi:10.1242/dev.200761)
Supplement: Supplementary information [file develop-149-200761-s1.pdf]

Figure S1

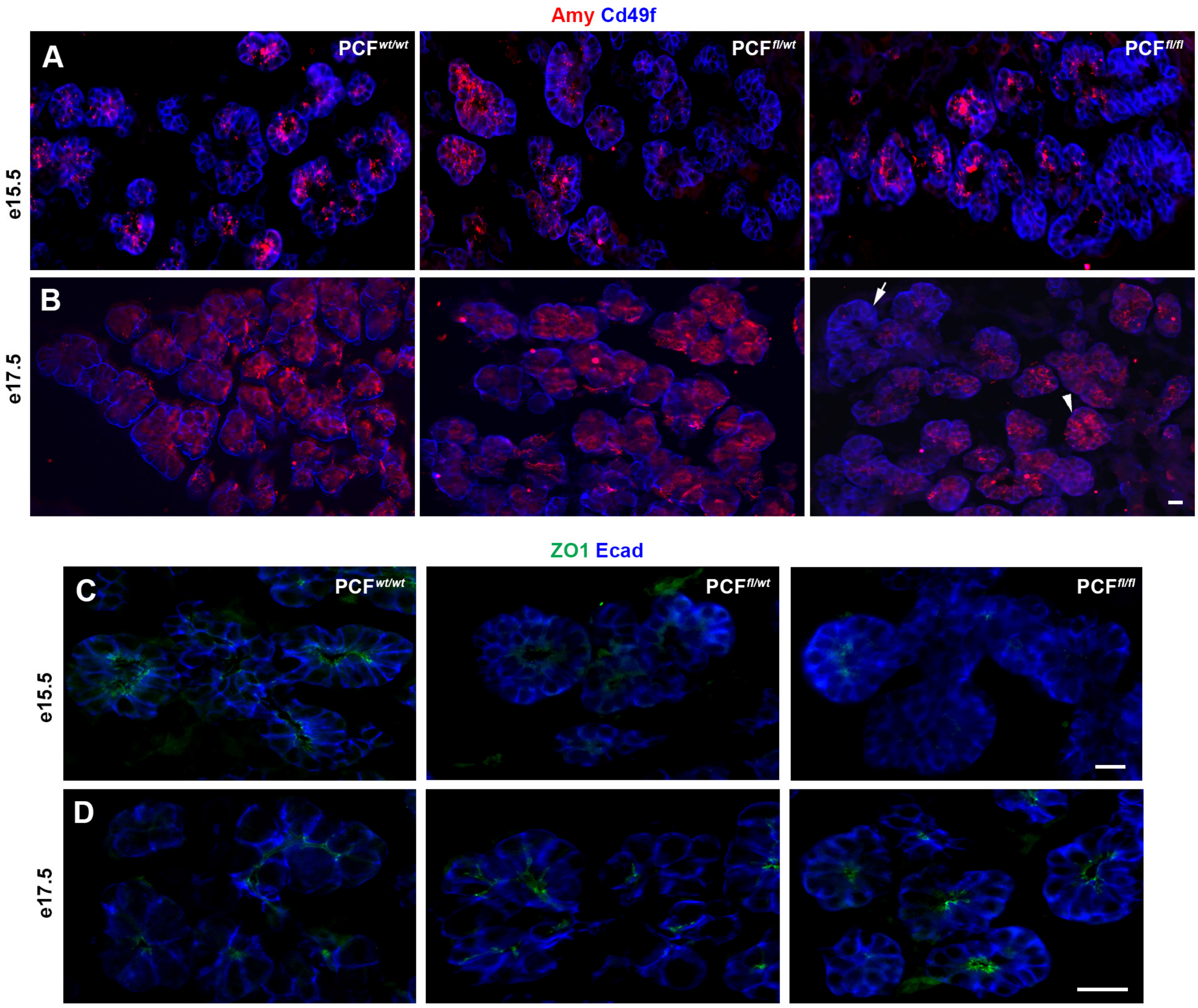

Fig. S1. Delayed acinar differentiation is observed in the PdxCre;*Fak*$^{fl/fl}$ pancreas. (A-D) Immunostaining of sections obtained from E15.5 (A, C) PCF$^{wt/wt}$ (n=4), PCF$^{fl/wt}$ (n=3) or PCF$^{fl/fl}$ (n=3) or E17.5 (B, D) PCF$^{wt/wt}$ (n=4), PCF$^{fl/wt}$ (n=4) or PCF$^{fl/fl}$ (n=4) pancreas for detection of amylase and Cd49f (A, B), or Ecadherin and ZO1 (C, D) showing delayed acinar differentiation in the PCF$^{fl/fl}$ pancreas. Arrow in (B) highlights amylase^{-} cluster with basolateral distribution of Cd49f. Arrowhead in (B) marks an acinar cluster expressing amylase with basal Cd49f localization.

Figure S2

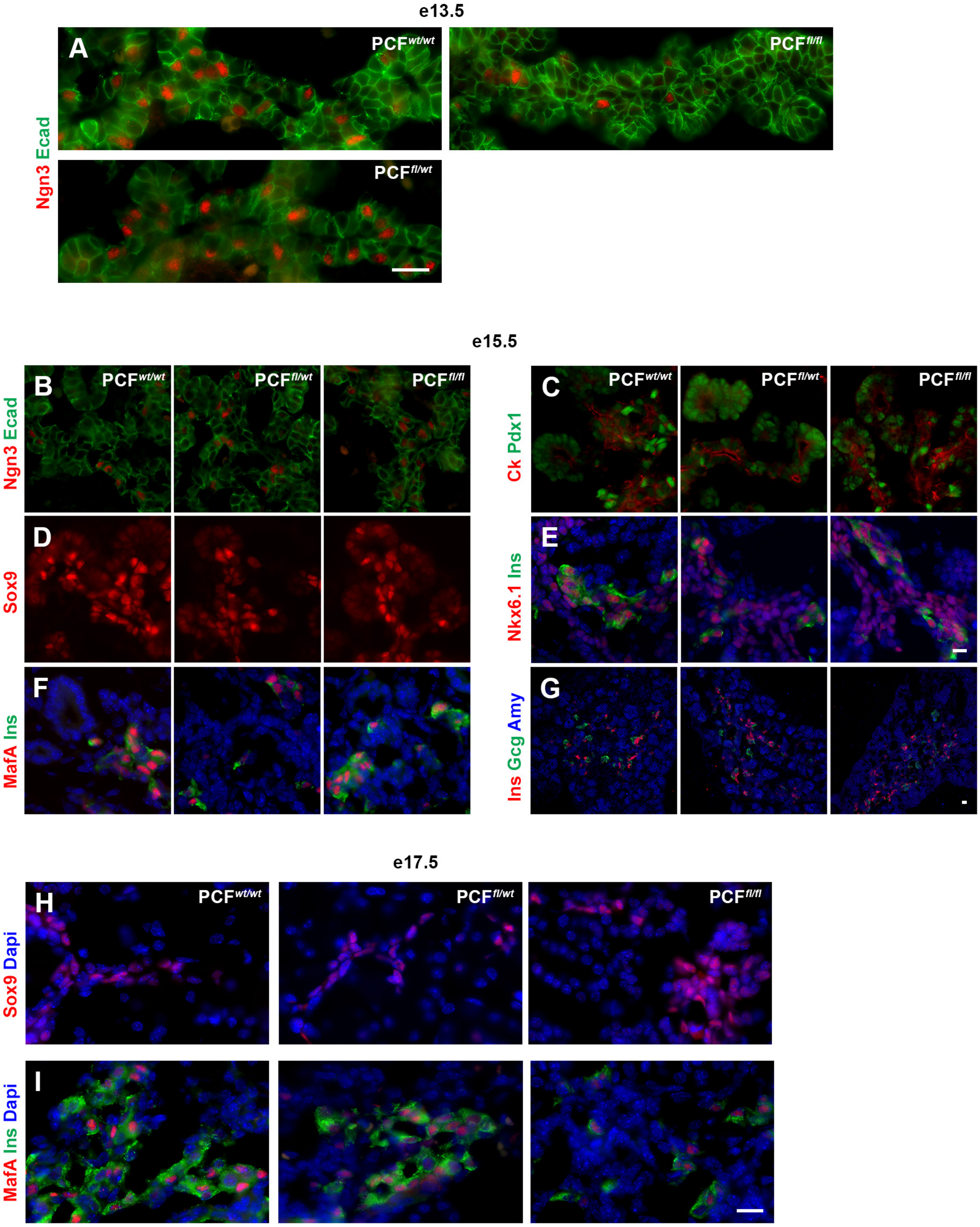

Fig. S2. Pancreatic endocrine differentiation is delayed in the PdxCre;*Fak*$^{fl/fl}$ embryos. (A-I) Immunostaining of sections obtained from e13.5 (A), e15.5 (B-G) PCF$^{wt/wt}$ (n=4), PCF$^{fl/wt}$ (n=3) or PCF$^{fl/fl}$ (n=3) or E17.5 (H, I) PCF$^{wt/wt}$ (n=4), PCF$^{fl/wt}$ (n=4) or PCF$^{fl/fl}$ (n=4) pancreas for detection of E-cadherin and Ngn3 (A, B), Pdx1 and Cytokeratin (C), Sox9 (D, H), insulin and Nkx6.1 (E), insulin and MafA (F, I), or glucagon, insulin and amylase (H). Scale bars 20 µm.

Development • Supplementary information

Figure S3

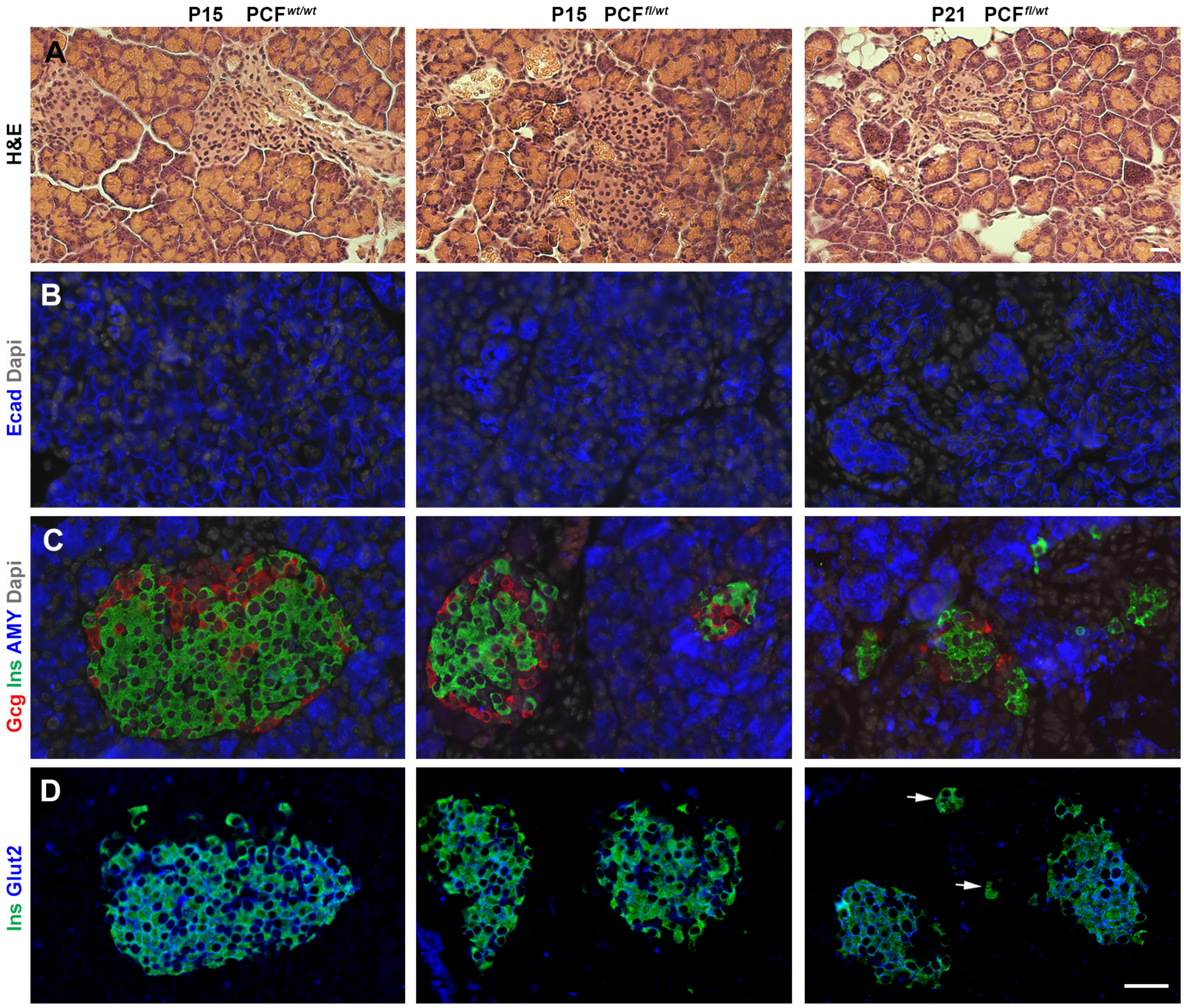

Fig. S3. Abnormal postnatal pancreatic tissue architecture in P21 PdxCre;FAK$^{fl/wt}$ mice. (A-D) H&E staining (A) or immunostaining of PCF$^{wt/wt}$ (n=5), or PCF$^{fl/wt}$ (n=5) postnatal days 15 or 21 pancreas for detection of E-cadherin (B), insulin, glucagon and amylase (C), or insulin and Glut2 (D). Note the early signs of periductal stromal response and loss of the lobular acinar parenchyma in the P21 PCF$^{fl/wt}$ pancreas in (A, B). Arrows in (D) mark insulin^{+}/ GLUT2^{-} cells. Scale bars 20 µm.

Development • Supplementary information

Figure S4

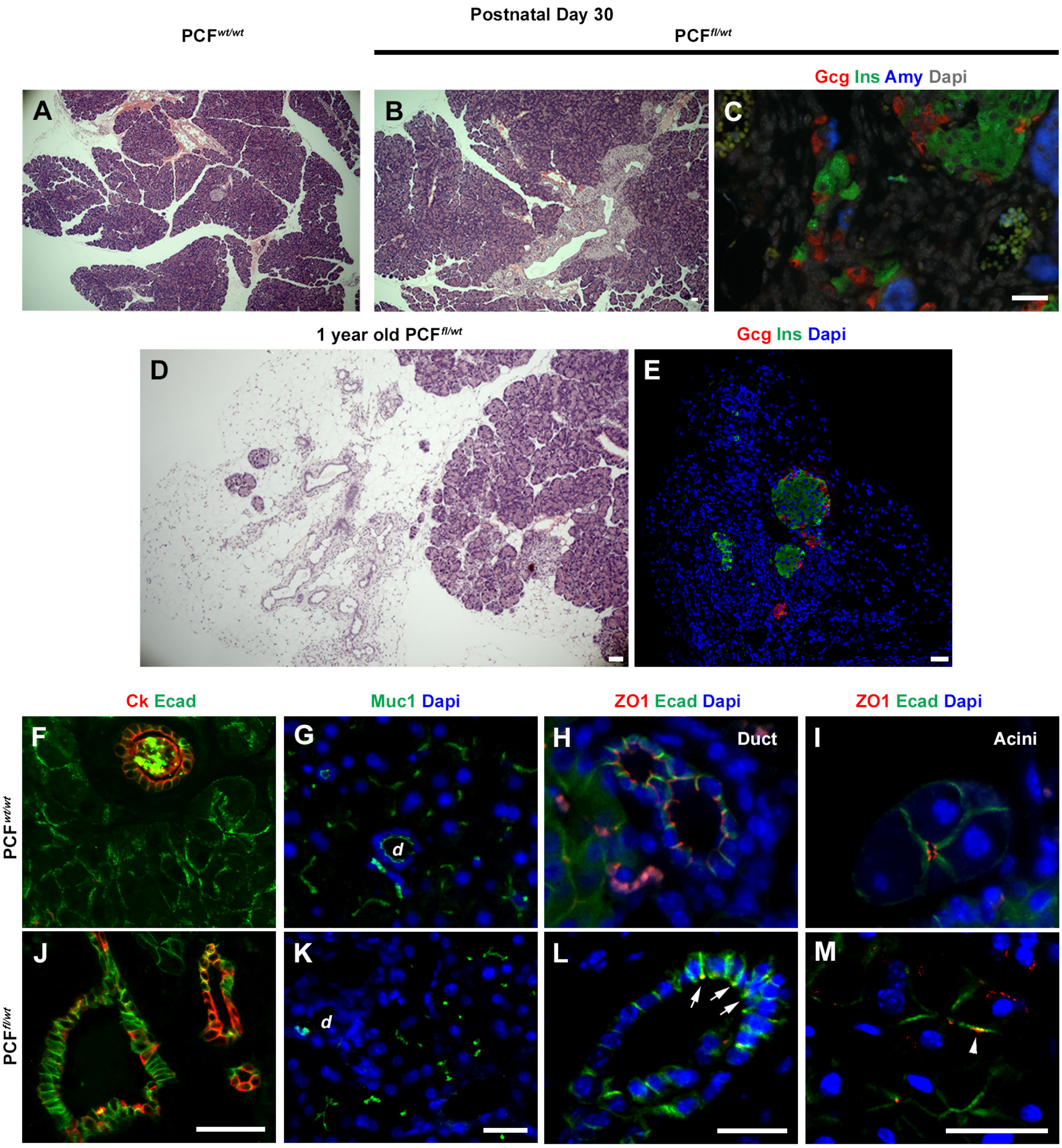

Development • Supplementary information

Fig. S4. Gradual acinar atrophy in the adult PdxCre;FAK$^{fl/wt}$ mice. (A, B) H&E of PCF$^{wt/wt}$ (n=3) (A) or PCF$^{fl/wt}$ (n=5) (B) postnatal day 30 pancreas showing endocrine islets in close proximity to the larger ducts. (C) Immunostaining of PCF$^{fl/wt}$ postnatal day 30 pancreas for detection of insulin, glucagon and amylase. (D, E) H&E (D) or immunostaining (E) for detection of insulin and glucagon on sections obtained from 1-year old PCF$^{fl/wt}$ mice (n=4) displaying acinar atrophy. (F-M) Immunostaining of PCF$^{wt/wt}$ (n=3) (F-I) or PCF$^{fl/wt}$ (n=5) (J-M) postnatal day 30 pancreas for detection of insulin, E-cadherin and cytokeratin (F, J), Muc1 (G, K), E-cadherin and ZO1 (H, I, M, M). Arrows in (L) mark few ductal cells in the PCF$^{fl/wt}$ pancreas with apical ZO1 localization. Arrow in (M) highlights a mutant acinar cell with lateral distribution of ZO1. *d*:duct. Scale bars 20 μm

Figure S5

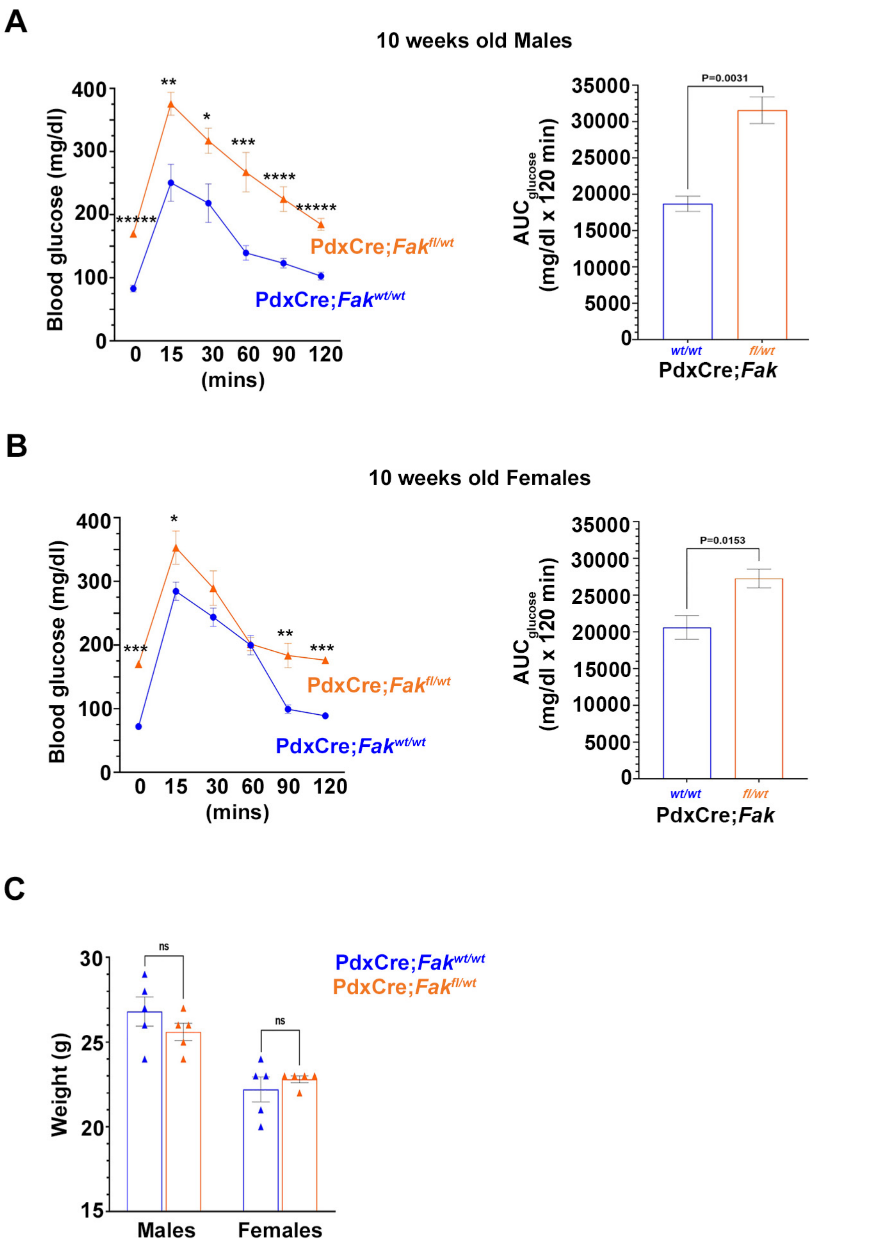

Development • Supplementary information

Fig. S5. Impaired β-cell function in the adult PdxCre;FAK$^{fl/wt}$ mice. (A, B) Glucose tolerance testing performed on 10-week old PCF$^{wt/wt}$ (n=5) and PCF$^{fl/wt}$ (n=5) males (A) or PCF$^{wt/wt}$ (n=5) and PCF$^{fl/wt}$ (n=5) females (B) showing impaired glucose clearance in the PCF$^{fl/wt}$ mice. (C) Body weights of 10-week old PCF$^{wt/wt}$ (5 males, 5 females) and PCF$^{fl/wt}$ (5 males, 5 females) mice did not show any differences between the cohorts. *p=0.0263, **p=0.0066, ***p<0.001, ****p=0.005, ****p=0.0013, *****p<0.0001 in (A) and *p=0.05, **p=0.0032, ***p<0.0001 in (B). Unpaired Student's t-test (A, B line graphs), Dunnett's multiple comparisons test (A, B bar graphs) and a Bartlett's test for equal variance (C). Data are mean ± standard error (SE).
